# Enhancing diffuse correlation spectroscopy pulsatile cerebral blood flow signal with near-infrared spectroscopy photoplethysmography

**DOI:** 10.1117/1.NPh.10.3.035008

**Published:** 2023-09-06

**Authors:** Kuan Cheng Wu, Alyssa Martin, Marco Renna, Mitchell Robinson, Nisan Ozana, Stefan A. Carp, Maria Angela Franceschini

**Affiliations:** a Massachusetts General Hospital, Athinoula A. Martinos Center for Biomedical Imaging, Charlestown, Massachusetts, United States; b Boston University, Department of Biomedical Engineering, Boston, Massachusetts, United States

**Keywords:** cerebral blood flow, cerebrovascular resistance, critical closing pressure, diffuse correlation spectroscopy, near-infrared spectroscopy

## Abstract

**Significance:**

Combining near-infrared spectroscopy (NIRS) and diffuse correlation spectroscopy (DCS) allows for quantifying cerebral blood volume, flow, and oxygenation changes continuously and non-invasively. As recently shown, the DCS pulsatile cerebral blood flow index (${\mathrm{pCBF}}_{i}$) can be used to quantify critical closing pressure (CrCP) and cerebrovascular resistance (${\mathrm{CVR}}_{i}$).

**Aim:**

Although current DCS technology allows for reliable monitoring of the slow hemodynamic changes, resolving pulsatile blood flow at large source–detector separations, which is needed to ensure cerebral sensitivity, is challenging because of its low signal-to-noise ratio (SNR). Cardiac-gated averaging of several arterial pulse cycles is required to obtain a meaningful waveform.

**Approach:**

Taking advantage of the high SNR of NIRS, we demonstrate a method that uses the NIRS photoplethysmography (NIRS-PPG) pulsatile signal to model DCS ${\mathrm{pCBF}}_{i}$, reducing the coefficient of variation of the recovered pulsatile waveform (${\mathrm{pCBF}}_{i\text{-}\text{fit}}$) and allowing for an unprecedented temporal resolution (266 Hz) at a large source-detector separation (>3  cm).

**Results:**

In 10 healthy subjects, we verified the quality of the NIRS-PPG ${\mathrm{pCBF}}_{i\text{-}\text{fit}}$ during common tasks, showing high fidelity against ${\mathrm{pCBF}}_{i}$ ($R^{2}$ 0.98±0.01). We recovered CrCP and ${\mathrm{CVR}}_{i}$ at 0.25 Hz, >10 times faster than previously achieved with DCS.

**Conclusions:**

NIRS-PPG improves DCS ${\mathrm{pCBF}}_{i}$ SNR, reducing the number of gate-averaged heartbeats required to recover CrCP and ${\mathrm{CVR}}_{i}$.

## 1 Introduction

Near-infrared spectroscopy (NIRS) is an established non-invasive diffuse optical method used to quantify hemoglobin oxygen saturation (${\mathrm{SO}}_{2}$) and hemoglobin concentration (Hb) changes from light attenuation at different wavelengths.^1^^,^^2^ Diffuse correlation spectroscopy (DCS) is a more recent optical technology that uses speckle fluctuations to measure an index for blood flow (${\mathrm{BF}}_{i}$). Specifically, DCS measures the intensity temporal autocorrelation function ($g_{2}$), which characterizes the displacement dynamics of scattering particles in the sample under interrogation.^3^^–^^5^ Combining DCS with NIRS allows for calculating the cerebral metabolic rate of oxygen^6^ and furthering our understanding of the relationship between hemoglobin concentration and cerebral blood flow (CBF) changes in healthy^7^^,^^8^ and pathological conditions.^9^^,^^10^ More recently, our and other groups have proposed the use of DCS pulsatile CBF index signals (${\mathrm{pCBF}}_{i}$) to quantify intracranial pressure (ICP), critical closing pressure (CrCP), pulsatility index (PI), and cerebrovascular resistance index (${\mathrm{CVR}}_{i}$) continuously and non-invasively.^11^^–^^16^ Despite the encouraging results, the low signal-to-noise ratio (SNR) of current DCS devices limits ${\mathrm{pCBF}}_{i}$ to source-detector separations (SDsep) of up to 2.5 cm, which reduces brain sensitivity in adults,^17^ and to achieve sufficient time-points within a pulsatile waveform, it requires cardiac-gated averaging of 50 arterial pulses,^11^ which dampens the pulsatile peaks and provides CrCP and ${\mathrm{CVR}}_{i}$ estimates at 0.02 to 0.07 Hz, rates that are too low to investigate the dynamic pressure-flow relationship of the cerebral vasculature.^18^ To overcome the DCS noise, increase SDsep, and recover ${\mathrm{pCBF}}_{i}$ with much less averaging, we propose a new method based on the combination of NIRS and DCS pulsatile signals.

Because NIRS measurements at the same sampling rate typically detect multiple orders of magnitude more photons, the SNR of NIRS is much better than the SNR of DCS,^19^^,^^20^ allowing for the measurement of pulsatile blood volume waveforms with high temporal resolution at long SDsep (≥3  cm).^21^ In particular, we recently developed an open-source, wearable, and wireless NIRS device, called FlexNIRS, with a low noise equivalent power (NEP<70  fW/√Hz), able to acquire 10 channels at up to 266 Hz sampling rate.^22^ The high SNR performance of this device allows us to resolve the pulsatile blood volume and its time derivative to perform cerebral pulse waveform analysis (PWA) on the pulsatile light absorbance of NIRS photoplethysmography (PPG) at a 3.3 cm SDsep (NIRS-PPG) with zero to a few beats averaging. PWA generally refers to the study of the morphology of the PPG waveform measured at short SDsep with pulse oximetry devices.^23^ Morphological features extracted from the superficial PPG and its time derivative have been investigated in the literature and often include amplitude, latency, and width of the PPG wave contour. These features are generally located with algorithms that find the local maxima and minima in the signal and its first to third time derivatives.^23^ PWA quantifies pulse wave characteristics to obtain information on the cardiovascular state, and correlations of specific features with skin blood vessel aging, stiffness, and peripheral resistance have been demonstrated.^24^^–^^27^ The ability to measure long SDsep PPG and its time derivative extends the analysis to characterize cerebral blood vessels and opens a new dimension to studying brain health with diffuse optical methods.^28^^–^^30^ Further, when simultaneously measuring with DCS and NIRS, by exploiting the pulsatile blood volume and blood flow relationship,^31^^–^^33^ we can separate the pulsatile inflow and outflow and model ${\mathrm{pCBF}}_{i}$ as a linear contribution of NIRS-PPG and its first time derivative [d(NIRS-PPG)/dt]. The resulting fitted ${\mathrm{pCBF}}_{i\text{-}\text{fit}}$ displays an exceeding SNR over DCS, while accurately matching DCS pulsatile flow, allowing us to estimate PI, CrCP, and ${\mathrm{CVR}}_{i}$ at the cardiac frequency.

To validate this model, we measured 12 healthy subjects simultaneously with the FlexNIRS and a state-of-the-art DCS prototype available in our lab, which operates at 1064 nm and employs superconducting nanowire single-photon detectors (SNSPD). The SNSPD-DCS system provides a >16-fold SNR increase versus the standard DCS technology,^17^ allowing us to resolve ${\mathrm{pCBF}}_{i}$ at larger separations and to use lower cardiac-gated averaging. We performed the NIRS and DCS measurements on the subjects while performing standard tasks that change cerebral and systemic physiology and recovered both pulsatile and slow varying signals under various conditions.

## 2 Material and Methods

### 2.1 Participants and Measurement Protocol

For the study, we enrolled and measured 12 healthy subjects. Two subjects were excluded due to low DCS data quality: low DCS photon counts (<20  kcps/s) and a low coherence factor (Siegert relationship β<0.3).^34^ Data from these two subjects were not included in this paper and were not numbered. The remaining 10 subjects included 6 males and 4 females of diverse skin tones, with an age range of 21 to 68 years old (Table S1 in the Supplementary Material). The study protocol was reviewed and approved by the Mass General Brigham Institutional Review Board (IRB No. 2020P002463) in accordance with Ethical Principles and Guidelines for the Protection of Human Subjects (Belmont Report). All subjects signed the informed consent before participating in the study.

The protocol consisted of cold pressor tests (CPTs), paced hyperventilation (HV), and brief repeated breath-holding (BH) tasks. For the CPT, subjects submerged one hand in warm water (30°C to 36°C) for 2 minutes; then they moved the hand into icy cold water (3°C to 5°C) for 1 min; finally, they moved the hand back into the warm water for 2 more min. CPT is known for raising blood pressure,^35^ and the protocol barred any subject with high blood pressure from performing this task. For the paced HV, after a 1-minute baseline of sale-paced breathing, subjects breathed at a rate of 70 breaths per minute following a metronome cue for a minute and then resumed their self-paced breathing for two more minutes. For the BH task, after a 1-minute baseline, subjects repeated a set of 20 s BH and 40 s normal breathing for four times, ending with an extended recovery period of 1 min.

### 2.2 Devices

**Fig. 1:**
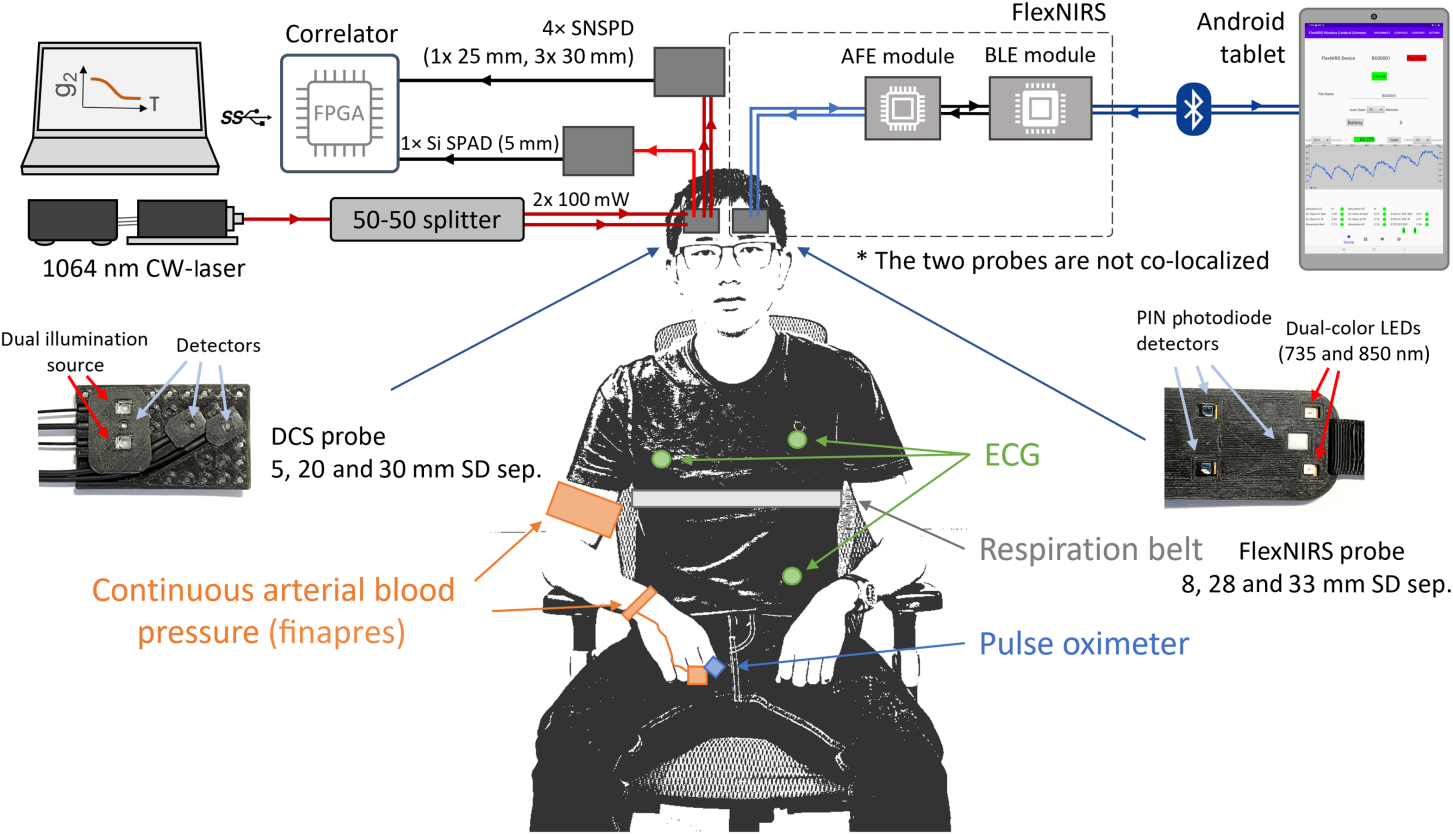
Measurement setup. Right: FlexNIRS for hemoglobin concentration and ${\mathrm{SO}}_{2}$. A piece of white electrical tape was applied to the short SDsep PD to attenuate the light. Left: SNSPD-based DCS for longer separations (2.0 and 3.0 cm) and Si SPAD-based DCS for the short separation (0.5 cm). Middle: pictures of the FlexNIRS and DCS probes. Bottom: the physiological signals from BIOPAC and Finapres recorded with PowerLab.

The wearable NIRS system used in the study is a continuous wave, open-source, low-cost, and wearable wireless cerebral oximeter called FlexNIRS, recently developed in our group.^22^ It consists of a wearable NIRS headband with an embedded flexible optical probe that communicates with a tablet through Bluetooth low energy (BLE) and is powered by a rechargeable lithium-ion battery (Fig. 1). The probe includes two dual-wavelength LEDs (735 and 850 nm, SMT735D/850D, Marubeni, Japan) and three PIN-photodiodes (VEMD5060X01, Vishay Intertechnology, Inc., United States), forming two short SDsep channels of 0.8 cm and two pairs of symmetrically arranged long SDsep channels of 2.8 and 3.3 cm. This geometry allows for a self-calibrating multi-distance scheme that uses two values at each separation to eliminate the effect of different component efficiencies and therefore provides a robust measurement of cerebral hemoglobin oxygenation without the need for external calibration.^36^^,^^37^ The use of surface-mount source and detector components in direct contact with the skin minimizes signal attenuation, and the use of a dedicated analog front-end chip that amplifies and digitizes the signals on one integrated circuit results in a device with low NEP (<70  fW/√Hz). The optimized custom software allows for a sampling rate of 266.66 Hz for all channels. This was a >2.5 times improvement in the sampling rate over the original description of the FlexNIRS device (100 Hz).^22^ This was achieved through shortening the sampling durations (LED-on time for all was reduced from 366 to 117  μs) and eliminating repeated ambient and short SDsep channels.

The DCS system used in this study operates at 1064 nm using a long coherence length laser (CrystaLaser, United States) and four high photon detection efficiency (88%) SNSPD detectors (Opus One, Quantum Opus, United States)^17^ (Fig. 1). To further increase SDsep while maintaining SNR, we used a dual-source illumination scheme, splitting the laser into two 3.5 mm diameter illumination spots, each emitting up to the maximal permissible exposure limit of 103 mW at 1064 nm (ANSI Z136.1). Source and detector fibers were terminated with prisms into a custom optical probe made of flexible 3D-printing material (NinjaTek, United States) and arranged to form SDsep of 2.0 cm (one detector fiber) and 3.0 cm (three detector fibers) (Fig. 1). In addition, to acquire scalp signals, we used a silicon single-photon avalanche diode (Si SPAD) detector at 0.5 cm from the sources. A custom 20-channel field-programmable gate array board documented the arrival time of each photon at 150 MHz resolution, and a custom data acquisition software calculated and displayed the $g_{2}$ and the blood flow index for each channel in real time.

To avoid possible crosstalk between FlexNIRS and the DCS, we positioned the two probes symmetrically on opposite sides of the forehead with the source placed laterally to maximize the distance between them.

We monitored systemic physiology using a non-invasive continuous arterial blood pressure (ABP) monitor (Finapres Medical Systems, Netherlands; Finapres NOVA) and BIOPAC modules (BIOPAC Systems Inc., United States) for electrocardiography (ECG; ECG100), pulse oximetry peripheral oxygen saturation (${\mathrm{SpO}}_{2}$; OXY100E), and respiratory rate (SS5LB). All systemic physiological signals were acquired with a 1000 Hz digital data acquisition device (ADInstruments, New Zealand; PowerLab).

### 2.3 Data Analysis

FlexNIRS and DCS data were analyzed using standard routines to recover slow varying hemodynamic changes and with cardiac-gated averaging algorithms to extract pulsatile waveforms.

#### 2.3.1 Slow varying signals

To quantify slow changes in ${\mathrm{SO}}_{2}$ and hemoglobin concentration, we used a mixed approach based on the multi-distance self-calibrating method described in Ref. 22 to calculate initial values and a single-distance modified Beer–Lambert law method to calculate hemoglobin changes. After ambient light subtraction, followed by a 1-s moving averaging, light intensity attenuation at 2.8 and 3.3 cm was used to recover absorption coefficients ($\mu_{a}$), assuming reduced scattering coefficients (${\mu_{s}}^{\prime }$) of $6.8\;{\mathrm{cm}}^{-1}$ at 735 nm and $5.9\;{\mathrm{cm}}^{-1}$ at 850 nm.^22^^,^^38^ A 75% water fraction was used to estimate hemoglobin concentration and oxygenation.^39^ Changes in ${\mathrm{SO}}_{2}$ and hemoglobin concentration with respect to the initial values were calculated for each subject by considering each source–detector pair independently. This hybrid method was used to eliminate spurious results of the multi-distance method due to tissue inhomogeneity (seeFig. S1 in the Supplementary Material).

To estimate slow varying blood flow changes, we computed the $g_{2}$ curves at 1 Hz smoothed with a moving average of 3 s for each SDsep. The $g_{2}$ obtained by three colocalized detectors at 3.0 cm were averaged together to increase SNR. We calculated ${\mathrm{BF}}_{i}$ by fitting the $g_{2}$ to the semi-infinite correlation diffusion equation,^40^ using optical properties ${\mu_{s}}^{\prime }$ of $4.7\;{\mathrm{cm}}^{-1}$ and $\mu_{a}$ of $0.18\;{\mathrm{cm}}^{-1}$. The scattering was extrapolated from the values used for FlexNIRS at shorter wavelengths to 1064 nm, and the absorption was obtained assuming typical hemoglobin and water concentrations.^41^

In addition, we calculated heart rate (HR) as the inverse of the R-R interval in the ECG signals and extracted the time series of ABP from calibrated Finapres measurements and ${\mathrm{SpO}}_{2}$ from the pulse oximeter. For group averaging, optical and systemic measured parameters were down-sampled to 1 Hz, subjected to a 3-s window moving average, and normalized with respect to the initial baseline. The four repetitions of BH were averaged together after detrending and normalizing the data to the three-second periods before the BH. Before averaging across subjects, the missing points that were rejected due to motion artifacts were interpolated.

#### 2.3.2 Pulsatile waveform analysis

The pulsatile waveform of NIRS-PPG was extracted from the 850 nm channels where the pulsatile signal amplitude is larger. A wavelength at the hemoglobin isosbestic point would be ideal to measure pulsatile blood volume. Because the arterial oxygenation is relatively constant, the use of non-isosbestic wavelengths is acceptable. In fact, assuming an arterial oxygenation drop of 10% from 100% to 90%, larger than the arterial oxygenation changes measured in this study, at 850 nm the amplitude of the pulsation changes by <3%. Moreover, in our analysis we only consider the relative amplitude of features of the pulsatile waveform, for which the wavelength dependence is further minimized as arterial oxygenation changes within a heartbeat are negligible.

To extract pulsatile waveforms from the FlexNIRS data, we first subtracted ambient light from the raw intensity measured at 266 Hz and removed data segments with motion artifacts as detected by the embedded accelerometer and gyroscope. We used a high pass filter ($f_{c}=0.4\;\mathrm{Hz}$) to remove breathing and Mayer waves and a low pass filter ($f_{c}=14\;\mathrm{Hz}$) to remove high-frequency noise. We then calculated the delta absorbance, or delta optical density ($\Delta \mathrm{OD}=\ln[I_{0}/I(t)]$, $I_{0}$: baseline light intensity)^42^ at 850 nm as NIRS-PPG. In addition to motion artifacts, we rejected distorted heartbeats that had outliers, which were defined as data points deviating from the median waveform by more than a 2.5 interquartile range (IQR). The median and IQR were calculated with cardiac-gated heartbeats within a short moving window of 15 s with a 50% overlap. To establish condition-representing NIRS-PPG waveforms, we gate-averaged heartbeats measured during the various tasks (conditions): each 1-minute baseline was considered to be one condition; the same was done for each recovery segment, excluding the first 20-s transition period; during the HV and CPT, we excluded the initial transitions and considered the last 40 s with more stable hemodynamic responses; and for the BH condition, we considered a period of 20 s centered around the end of the BH and averaged the four repetitions together. The FlexNIRS optical data were aligned with the ECG signals by matching the beat-to-beat HR variability. For the gate averaging, the cardiac cycles were identified using the diastolic onsets in the NIRS-PPG signals, and each heartbeat was normalized by stretching, through interpolation, to a uniform period equivalent to the subject average HR during that condition. We performed these calculations for both NIRS wavelengths and all source-detector pairs; for each subject NIRS-PPG PWA analysis, we report the 3.3 cm pair at 850 nm with the highest SNR ($\mathrm{SNR}=\sigma_{\text{pulsation}}/\sigma_{\text{noise}}$; σ: standard deviation). The SNR was calculated based on high-pass filtered light intensity signals ($f_{c}=0.4\;\mathrm{Hz}$): the pulsation is defined as the low-pass filtered ($f_{c}=14\;\mathrm{Hz}$) intensity, and the noise is the difference between pre and post low-pass filtering. In two subjects (Nos. 3 and 8), a 2.8 cm channel was used because the 3.3 cm channels had a low SNR (<15).

To obtain DCS pulsatile blood flow waveforms, we computed $g_{2}$ curves at 100 Hz using 40 ms of integration time, i.e., a 20 ms photon-inclusion radius. The data periods excluded in the FlexNIRS due to motion artifacts were also excluded for the DCS time series. Representative DCS pulsatile blood flow waveforms during the various conditions were calculated through cardiac-gated averaging of $g_{2}$ curves instead of the ${\mathrm{CBF}}_{i}$. Each heartbeat onset was determined by the co-acquired ECG R-peaks; across heartbeats, $g_{2}$ curves of the same location in the cardiac cycle were averaged together as done in a previous study.^11^ As for the slow ${\mathrm{CBF}}_{i}$, the gate-averaged $g_{2}$ curves were fitted using the semi-infinite correlation diffusion equation,^40^ assuming the optical properties listed previously. During the fitting iterations for the ${\mathrm{pCBF}}_{i}$, as systolic $g_{2}$ curves of high flow lack a clear plateau, β was kept constant. For each measurement, this value was set to be the median β from the traditional free-β fit.

Pulse waveform analysis was performed to quantify the peak amplitudes and their corresponding latencies of NIRS-PPG, d(NIRS-PPG)/dt, and ${\mathrm{pCBF}}_{i}$. We used a time derivative method^23^ modified for brain PPG. When comparing pulsatile NIRS and DCS, to match the NIRS sampling rate, ${\mathrm{pCBF}}_{i}$ was up-sampled to 266 Hz through cubic spline interpolation; to compensate for the longer integration time of DCS (40 ms), NIRS-PPG and d(NIRS-PPG)/dt were subjected to a moving average of 11 points (i.e., 11/266.66  Hz≈40  ms).

### 2.4 Modeling of ${\mathrm{pCBF}}_{i}$ as a Function of NIRS-PPG

Herein we examine the relationship between ${\mathrm{pCBF}}_{i}$ and NIRS-PPG when the two are simultaneously measured. The tissue is assumed to be a semi-infinite homogenous medium, and a similar depth sensitivity is assumed for the two methods. The pulsatile light absorbance of NIRS-PPG is proportional to the pulsatile blood volume changes. As a result, its first time derivative represents the changing rate of blood volume, which is the difference between the blood inflow and the blood outflow during a heartbeat and is given as^31^^–^^33^

$${\mathrm{BF}}_{\text{in}}-{\mathrm{BF}}_{\text{out}}=\gamma \frac{d}{dt}(\mathrm{NIRS}\text{-}\mathrm{PPG}).$$ (1)

As suggested by a prior study, if we assume the outflow blood drains out passively, ${\mathrm{BF}}_{\text{out}}$ is modeled to be proportional to the pulsatile volume of blood within the area interrogated by diffusely propagating photons in the tissue bed, which is given as^31^

$${\mathrm{BF}}_{\text{out}}=a(\mathrm{NIRS}\text{-}\mathrm{PPG})+b.$$ (2)

DCS ${\mathrm{pCBF}}_{i}$ represents the total flow through the vasculature, which gives

$${\mathrm{BF}}_{\text{in}}+{\mathrm{BF}}_{\text{out}}=k\cdot {\mathrm{pCBF}}_{i},$$ (3)

with k being the scaling factor that converts the DCS $p{\mathrm{CBF}}_{i}$ units ($\mathrm{cm}/s^{2}$) to the blood flow units [mL/(100  g*min)] for perfusion per tissue volume. DCS ${\mathrm{BF}}_{i}$ measures the motion of red blood cells, which is proportional to blood flow, as shown in previous validation studies^43^ and recent modeling studies.^44^^,^^45^

By combining these equations, we derive ${\mathrm{pCBF}}_{i\text{-}\text{fit}}$, the estimated ${\mathrm{pCBF}}_{i}$ based on the linear combination of the NIRS-PPG signal and its derivative, as

$${\mathrm{pCBF}}_{i\text{-}\text{fit}}=\frac{\gamma }{k}\frac{d}{dt}(\mathrm{NIRS}\text{-}\mathrm{PPG})+\frac{2a}{k}(\mathrm{NIRS}\text{-}\mathrm{PPG})+\frac{2}{k}b\;=C_{1}\frac{d}{dt}(\mathrm{NIRS}\text{-}\mathrm{PPG})+C_{2}(\mathrm{NIRS}\text{-}\mathrm{PPG})+C_{3}.$$ (4)

An additional parameter, the alignment lag $C_{0}$ was applied to temporally align the signals acquired with independent devices. This is due to the uncertainty of absolute time bases of the devices introduced by the BLE communication and the clocks of the tablet and the computer. A non-linear fit, Levenberg-Marquardt, was performed to minimize the least-squares of residuals and determine the four parameters ($C_{0}$, $C_{1}$, $C_{2}$, and $C_{3}$). We found that this approach results in many local minima, to which, based on the initial guess, non-linear fitting algorithms are known to converge, as shown in Ref. 46. Hence, we used an initial guess grid search for Levenberg–Marquardt to find the proper initial guess, which leads to the convergence to the global minimum.

Finally, by combining Eqs. (1), (3), and (4), we were able to separate the inflow (${\mathrm{BF}}_{\text{in}}$) and outflow (${\mathrm{BF}}_{\text{out}}$) contributions to the pulsatile flow, given as

$${\mathrm{BF}}_{\text{in}}=\frac{k}{2}({\mathrm{pCBF}}_{i}+C_{1}\frac{d}{dt}(\mathrm{NIRS}\text{-}\mathrm{PPG})),$$ (5)

$${\mathrm{BF}}_{\text{out}}=\frac{k}{2}({\mathrm{pCBF}}_{i}-C_{1}\frac{d}{dt}(\mathrm{NIRS}\text{-}\mathrm{PPG})).$$ (6)

We calculated $C_{1}$ and $C_{2}$ for each task condition by fitting the averaged ${\mathrm{pCBF}}_{i}$ and NIRS-PPG signals and then projected these parameters back to NIRS-PPG to estimate ${\mathrm{pCBF}}_{i\text{-}\text{fit}}$ with no cardiac-gated averaging. $C_{1}$ and $C_{2}$ were interpolated during task transitions. $C_{3}$ was replaced by the slow varying mean ${\mathrm{CBF}}_{i}$ time traces calculated above. We evaluated the SNR improvement with respect to SNSPD-DCS ${\mathrm{pCBF}}_{i}$, by calculating the coefficient of variation (CV) as σ/μ (σ: standard deviation and μ: mean) of each point along the cardiac cycle across heartbeats during the 60-s baselines.

### 2.5 Critical Closing Pressure, Pulsatility Index, and Cerebrovascular Resistance

We obtained the PI, CrCP, and ${\mathrm{CVR}}_{i}$ using the ${\mathrm{pCBF}}_{i}$ and the alternative NIRS-PPG-reconstructed ${\mathrm{pCBF}}_{i\text{-}\text{fit}}$ with much less cardiac-gated averaging. With the SNSPD-DCS-only ${\mathrm{pCBF}}_{i}$, due to its higher noise, we used 20 heartbeats; with NIRS-PPG-reconstructed ${\mathrm{pCBF}}_{i\text{-}\text{fit}}$, we used 4 heartbeats. The signals were aligned with the pulsatile ABP (pABP) obtained from the Finapres using the same cardiac-gated averaging. CrCP was obtained by linearly extrapolating the ${\mathrm{pCBF}}_{i}$ versus pABP relationship to the pABP-axis intercept. Because of the non-linearities during the systolic phase, only the diastolic run-off phase of the pulse cycle was considered, as done previously.^11^ In the past, the dicrotic notch (DN) was manually found for each subject; however, now due to the high SNR of the ${\mathrm{pCBF}}_{i\text{-}\text{fit}}$, we can automatically find the diastolic run-off using a PWA algorithm,^23^ which we customized for brain NIRS-PPG. The ${\mathrm{CVR}}_{i}$ was calculated as the inverse of the slope of the pulsatile pressure-flow relationship, which is given as

$${\mathrm{CVR}}_{i}=\frac{d(\mathrm{pABP})}{d({p\mathrm{CBF}}_{i})}.$$ (7)

We rejected outliers with loose criteria that only include data points that have pressure-flow regression $R^{2}$ of higher than 0.8, CrCP within −30 to 150 mmHg, and positive ${\mathrm{CVR}}_{i}$.

The PI, defined as the difference between the systolic and diastolic flow velocity divided by the mean flow velocity as measured by transcranial Doppler (TCD) ultrasound, is used to assess vascular resistance,^47^ intracranial pressure, and cerebral perfusion pressure.^48^ Herein we define PI of DCS as the difference between the systolic and diastolic ${\mathrm{pCBF}}_{i}$ divided by the mean. The same was calculated for ${\mathrm{pCBF}}_{i\text{-}\text{fit}}$.

When comparing results between conditions, after excluding outliers (exceeding 1.5 IQR above Q3 or below Q1), we examined normality with the Kolmogorov–Smirnov test (α=0.05). If normality was not found, instead of the pairwise t-test, we applied the paired-sample sign test.

## 3 Results

### 3.1 Pulsatile ${\mathrm{CBF}}_{i}$ and NIRS-PPG Waveforms

**Fig. 2:**
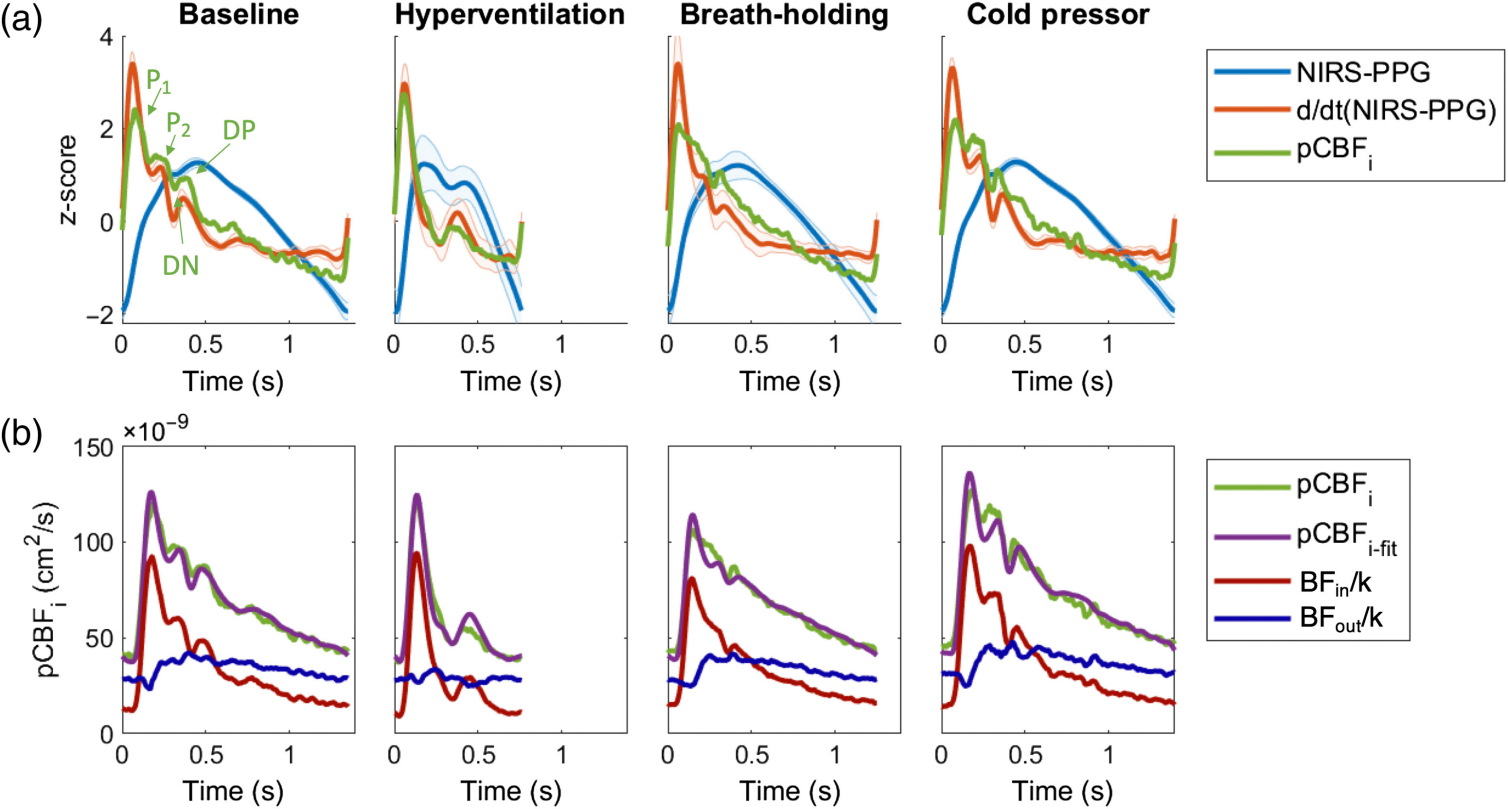
(a) Average pulsatile waveforms of a representative subject (No. 5) during the measured conditions. The features for PWA are listed on the baseline panel. (b) ${\mathrm{pCBF}}_{i}$ and ${\mathrm{pCBF}}_{i\text{-}\text{fit}}$ comparison and inflow and outflow contribution (in ${\mathrm{BF}}_{i}$ unit ${\mathrm{cm}}^{2}/s$) to the pulsatile flow on the same subject.

Cardiac-gated averaging of DCS and FlexNIRS signals was performed for each subject and condition. Examples of ${\mathrm{pCBF}}_{i}$, NIRS-PPG, and d(NIRS-PPG)/dt on a representative subject (No. 5) are shown in Fig. 2(a). The four panels show the pulsatile waveforms during baseline, HV, BH, and cold pressor conditions. We report the pulse waveforms in normalized z-scores, which is defined as the number of standard deviations away from the mean (calculated as $z=(x_{i}-\mu )/\sigma$. $x_{i}$: data points, μ: mean, and σ: standard deviation). We observed a strong similarity between ${\mathrm{pCBF}}_{i}$ and d(NIRS-PPG)/dt, with comparable morphology changes of the main peaks with different conditions. Across all subjects and conditions, the $R^{2}$ between ${\mathrm{pCBF}}_{i}$ and d(NIRS-PPG)/dt was 0.89±0.10.

**Fig. 3:**
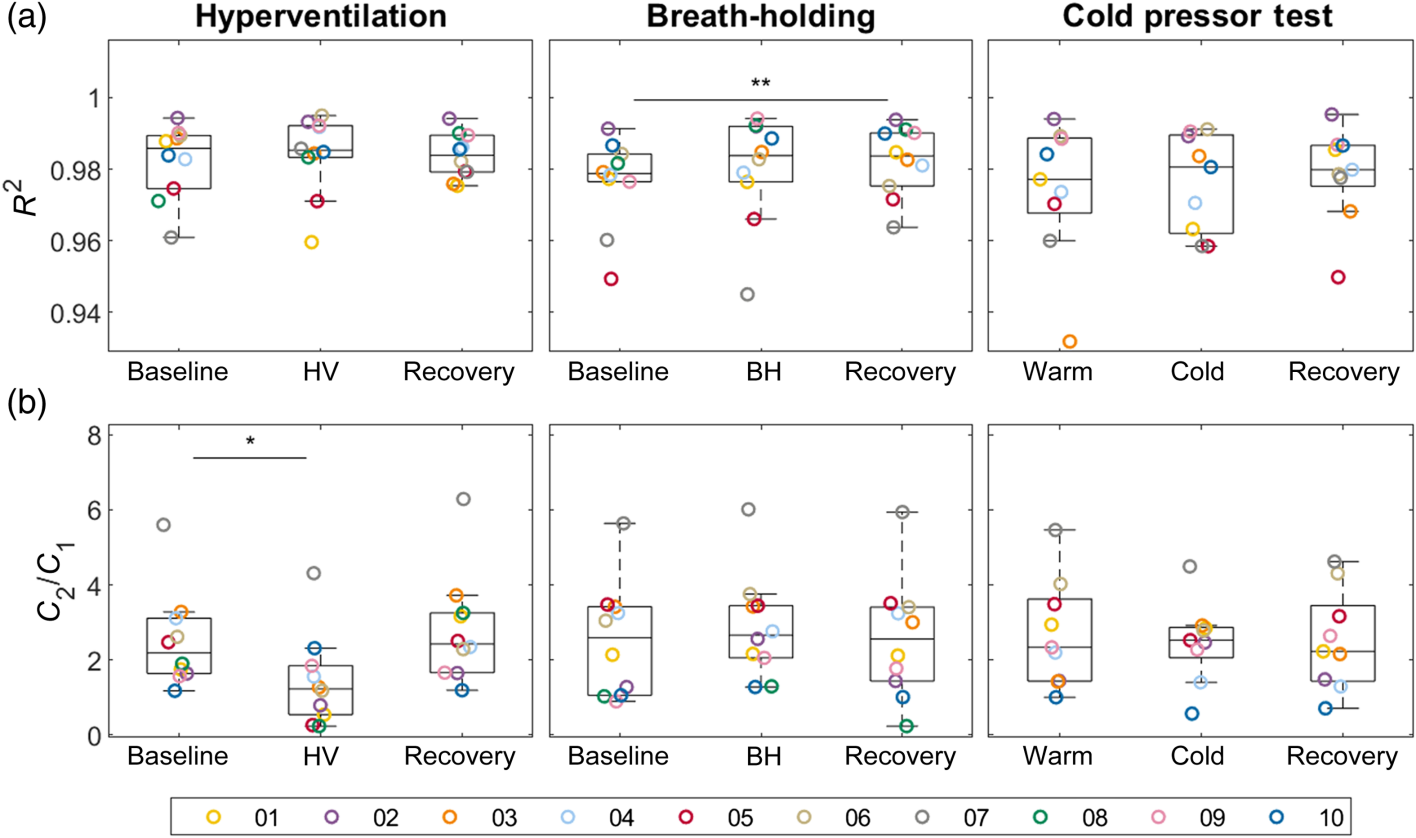
(a) Fit quality $R^{2}$ and (b) fitting parameters $C_{2}/C_{1}$ for the three tasks. Different colors represent different subjects. The asterisks indicate paired-sample sign test significant difference levels. (*: p<0.05, **: p<0.01, and ***: p<0.001)

The difference between ${\mathrm{pCBF}}_{i}$ and d(NIRS-PPG)/dt can be accounted for by incorporating the NIRS-PPG waveform contribution. Using Eqs. (4)–(6), we were able to reconstruct ${\mathrm{pCBF}}_{i}$ as ${\mathrm{pCBF}}_{i\text{-}\text{fit}}$ using NIRS-PPG and separate inflow and outflow [Fig. 2(b), results for the same representative subject]. We found ${\mathrm{pCBF}}_{i\text{-}\text{fit}}$ to be nearly identical to ${\mathrm{pCBF}}_{i}$, with $R^{2}$ of 0.98±0.01 across all subjects and conditions [Fig. 3(a)]. The fitting parameters $C_{1}$ and $C_{2}$ are reported in Fig. S2 in the Supplementary Material, and the ratio $C_{2}/C_{1}$ is shown in Fig. 3(b). The parameters $C_{1}$ and $C_{2}$ differ between subjects, but weakly depend on different conditions, except for HV, where $C_{1}$, $C_{2}$, and $C_{2}/C_{1}$ dropped (for $C_{2}/C_{1}$ relative to baseline, p<0.05, paired-sample sign test).

With the higher SNR of the ${\mathrm{pCBF}}_{i\text{-}\text{fit}}$, we could identify waveform features automatically. We quantified the systolic peak ($P_{1}$), secondary systolic peaks ($P_{2}$, tidal wave), DN and diastolic peak amplitudes, and time delay. As an example, the values of $P_{1}$, $P_{2}$, and $P_{2}/P_{1}$ across subjects and conditions are reported in Fig. S3 in the Supplementary Material. We found that $P_{2}/P_{1}$ dropped during HV (relative to recovery, p<0.01, paired-sample sign test) and increased during CPT (relative to baseline, p<0.01, paired-sample sign test).

### 3.2 Minimal Cardiac-Gated Averaging Noise Characterization

**Fig. 4:**
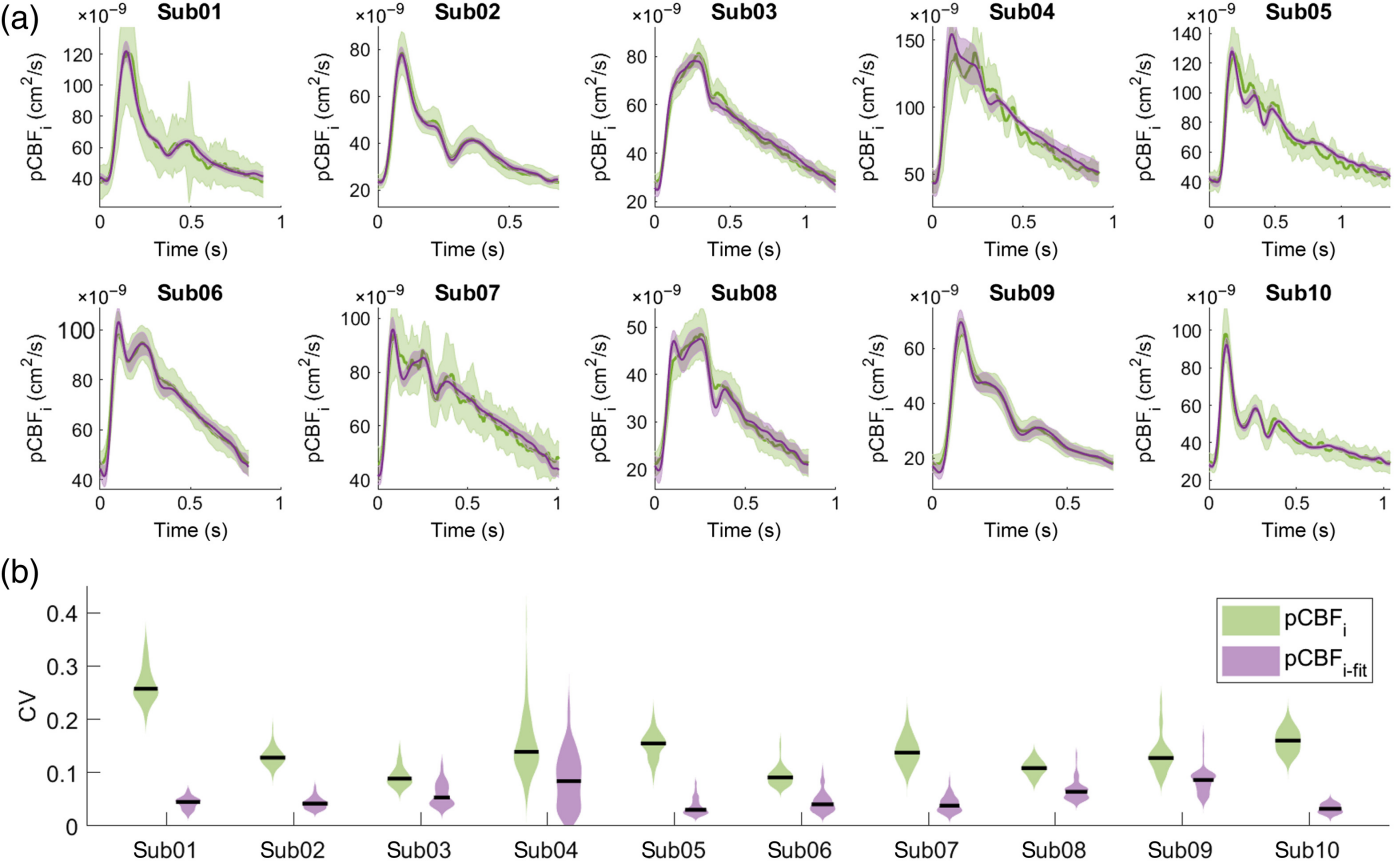
(a) SNSPD-DCS ${\mathrm{pCBF}}_{i}$ and NIRS-PPG ${\mathrm{pCBF}}_{i\text{-}\text{fit}}$ average and standard deviation of pulsatile signals during 60-s baseline. (b) Corresponding CV for the 10 subjects (violin plot).

To quantify the improvement in SNR of ${\mathrm{pCBF}}_{i\text{-}\text{fit}}$ with respect to SNSPD-DCS ${\mathrm{pCBF}}_{i}$, we evaluated the CV of the pulsatile waveforms during a 60-s baseline. Figure 4(a) shows the average and standard deviation of ${\mathrm{pCBF}}_{i}$ and ${\mathrm{pCBF}}_{i\text{-}\text{fit}}$ for each subject. The shapes of the pulsatile waveforms were diverse among subjects, yet the NIRS-PPG fits showed a strong agreement with the DCS-measured ${\mathrm{pCBF}}_{i}$. ${\mathrm{pCBF}}_{i\text{-}\text{fit}}$ provided an improvement on the CV, as the CV of ${\mathrm{pCBF}}_{i\text{-}\text{fit}}$ was consistently lower than the CV of ${\mathrm{pCBF}}_{i}$, and on average, ${\mathrm{CV}}_{\mathrm{pCBFi}}/{\mathrm{CV}}_{\mathrm{pCBFi}\text{-}\text{fit}}=3.1\pm 1.7$ [Fig. 4(b)]. The paired-sample sign test rejected the null hypothesis with p<0.01.

### 3.3 Slow Changes in Cerebral and Systemic Measured Parameters with Tasks

**Fig. 5:**
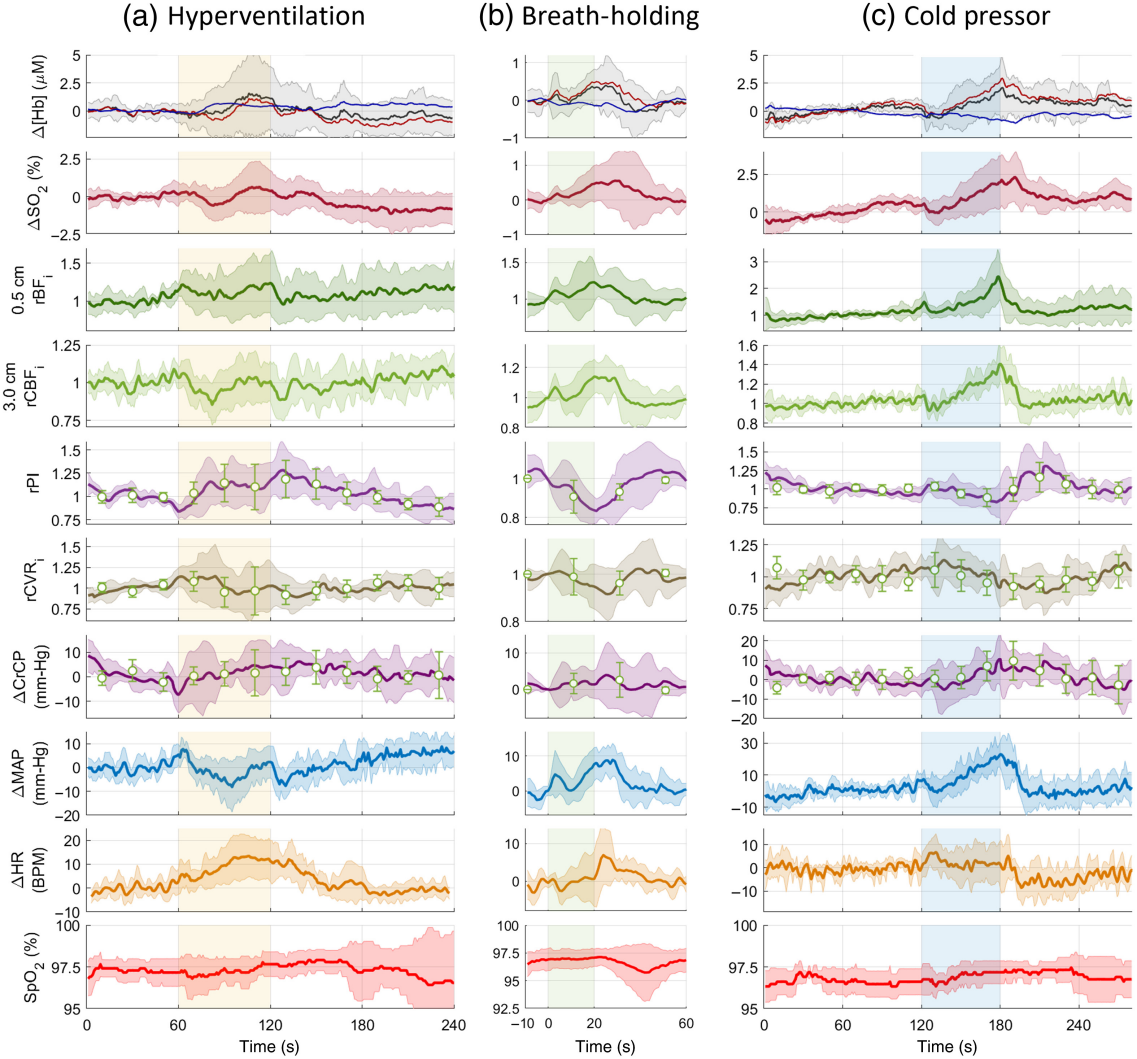
Group average of all measured parameters during (a) hyperventilation task (n=8, HV period in yellow), (b) BH task (n=8, block average of four repetitions, BH period in green), and (c) cold pressor test (n=7, CPT period in cyan). r-prefix indicates relative to, and Δ-prefix indicates delta changes; both are with respect to the average of 60-s initial baseline. The standard deviation across subjects is shown in lighter colors. rPI, ΔCrCP, and ${\mathrm{rCVR}}_{i}$ were calculated every 4 s using ${\mathrm{pCBF}}_{i\text{-}\text{fit}}$ (continuous trace) and every 20 s (circles) using ${\mathrm{pCBF}}_{i}$. All other parameters were calculated at 1 Hz with a 3 s moving average. For ΔHb graphs: red ΔHbO, blue ΔHbR, and black ΔHbT. The pulse amplitude of NIRS-PPG and its derivative are shown in Fig. S4 in the Supplementary Material.

In addition to the NIRS hemoglobin concentration and oxygenation changes and DCS relative changes of the CBF index during the three tasks, we calculated the PI, CrCP, and ${\mathrm{CVR}}_{i}$ changes with time using ${\mathrm{pCBF}}_{i}$ (one point every 20 s) and ${\mathrm{pCBF}}_{i\text{-}\text{fit}}$ (one point every 4 s). Across subjects, for the two methods, baseline CrCP was 32±13  mm-Hg and 36±14  mm-Hg, respectively, and baseline PI was 1.14±0.35 and 1.37±0.41, respectively. In Fig. 5, we show the slow cerebral responses to each task averaged across all subjects along with the systemic physiology responses, including ΔHb, $\Delta {\mathrm{SO}}_{2}$, scalp ${\mathrm{rBF}}_{i}$, ${\mathrm{CBF}}_{i}$, PI, ${\mathrm{CVR}}_{i}$, CrCP, mean ABP (MAP), HR, and ${\mathrm{SpO}}_{2}$. Subject No. 2 was excluded because of poor attachment of the FlexNIRS probe, which caused large light intensity jumps during task transitions. Subject No. 5 was excluded due to technical problems with the Finapres pABP measurement, which prevented us from calculating CrCP and ${\mathrm{CVR}}_{i}$. For the cold pressor task, subject No. 8 did not perform the task due to high blood pressure.

#### 3.3.1 Hyperventilation

HV causes hypocapnia due to over-breathing and is expected to cause vasoconstriction and a decrease in blood flow.^49^^,^^50^ Because cerebral metabolism is maintained during this period, the reduction in blood flow results in a decrease in ${\mathrm{SO}}_{2}$, which induces a vasoactive reaction to return the blood flow to the baseline level.^51^ This biphasic response was observed in HbT, ${\mathrm{SO}}_{2}$, and ${\mathrm{CBF}}_{i}$, whereas scalp blood flow (0.5 cm ${\mathrm{rBF}}_{i}$) showed minimal changes. The pulse amplitude of both NIRS-PPG and its time derivative increased steadily. A similar but smaller biphasic response was observed in ${\mathrm{CVR}}_{i}$ (opposite direction, i.e., initial increase and subsequent decrease), whereas PI and CrCP increased during HV and slowly returned to baseline during recovery. The observed systemic physiologic response to the HV was a significant increase in HR (13.5±8.6 BPM) and an initial increase (8±4  mmHg) and subsequent decrease (−8±11  mmHg) in MAP, as seen in Fig. 5(a).

#### 3.3.2 Breath-holding

A series of repeated brief breath holds caused an increase in blood pressure^52^^,^^53^ and a hypercapnic state.^54^^,^^55^ As shown in Fig. 5(b), the average response to a 20s BH task show increases in HbT (0.4±0.8  μM), ${\mathrm{SO}}_{2}$ (0.5%±0.8%), ${\mathrm{CBF}}_{i}$ (14%±11%), and scalp ${\mathrm{BF}}_{i}$ (23%±28%). The pulse amplitude of NIRS-PPG and its time derivative dropped. From the pulsatile parameters analysis, we observed a decrease in ${\mathrm{CVR}}_{i}$ and PI during BH. MAP increased (9±5  mmHg), whereas the HR remained relatively constant. ${\mathrm{SpO}}_{2}$ measured on the finger showed a drop (to ≤95%) in 4 out of 8 subjects. This drop was observed to lag behind the head and brain responses, with a nadir of about 22 s after the resumption of breathing. This delay of finger responses was expected and is consistent with literature findings.^56^

#### 3.3.3 Cold pressor test

The CPT induces both pain and a response of the autonomous nervous system, including an elevation in blood pressure.^35^ In our experiments, MAP increased substantially (23±8  mmHg), whereas HR and ${\mathrm{SpO}}_{2}$ remained relatively constant. After an initial small drop in HbT, ${\mathrm{SO}}_{2}$, and ${\mathrm{CBF}}_{i}$, the parameters increased throughout the remainder of the task. ${\mathrm{CBF}}_{i}$ increased by 41%±17%, whereas scalp ${\mathrm{BF}}_{i}$ reached a 143%±90% increase. The large increase in ${\mathrm{CBF}}_{i}$ and blood pressure caused a small increase in CrCP toward the end of the stimulus and a decrease in ${\mathrm{CVR}}_{i}$. PI showed a biphasic response: a decrease during CPT and a step increase when the hand was moved back into the warm water. The step increase was also observed in the pulse amplitude of both NIRS-PPG and its time derivative.

## 4 Discussion

We demonstrated that the simultaneous use of NIRS and DCS allows for the recovery of pulsatile blood flow waveforms with a higher SNR and a higher temporal resolution than with DCS alone, even when using a high-end DCS system such as the SNSPD-DCS at 1064 nm here. The use of a low noise, high sampling rate NIRS device, such as FlexNIRS (266 Hz), allowed for a three times improvement in ${\mathrm{pCBF}}_{i\text{-}\text{fit}}$ CV with respect to SNSPD DCS pCBFi (Fig. 4) and an increase in the sampling rate from 100 to 266 Hz at >3  cm SD separation. Moreover, the low noise of the recovered ${\mathrm{pCBF}}_{i\text{-}\text{fit}}$ allowed us to use automatic pulsatile feature extraction algorithms for PWA. This method was demonstrated with SNSPD DCS in 10 healthy subjects, showing that a three-parameter linear fitting model using the NIRS-PPG and its time derivative signals is sufficient to recover pCBFi with high accuracy ($R^{2}=0.98$) (Fig. 2). We showed that the fitting parameters are quite stable within the same subject, despite induced physiological changes, except for HV (Fig. 3). The changes in the fitting parameter $C_{2}$ during HV imply a change in the vasculature capacitance and resistance. Ideally, we should update the fitted parameters continuously during a monitoring session. This may be a potential issue when using a common DCS system, for which a much larger cardiac gated averaging is needed to provide a stable ${\mathrm{pCBF}}_{i}$ waveform, especially at 3 cm source-detector separations.

Because of the similarity of ${\mathrm{pCBF}}_{i}$ and d(NIRS-PPG)/dt ($R^{2}=0.89$), when performing PWA, if necessary, d(NIRS-PPG)/dt alone can be used to approximate ${\mathrm{pCBF}}_{i}$. Instead, to calculate CrCP, ${\mathrm{CVR}}_{i}$, and PI, the ${\mathrm{pCBF}}_{i}$ mean amplitude is needed to calibrate d(NIRS-PPG)/dt, so a combined NIRS-DCS approach is necessary. The use of ${\mathrm{pCBF}}_{i\text{-}\text{fit}}$ allows for calculating CrCP, ${\mathrm{CVR}}_{i}$, and PI with minimal averaging and detecting changes during transients. The 4 s average was done to reduce pulse-to-pulse variability and to obtain a more stable fitting when estimating CrCP. In the literature, ${\mathrm{CVR}}_{i}$ is often calculated using the mean CBF and the mean blood pressure values, which requires the assumption of a zero intercept on the pressure-flow relationship. This is not always the case as shown by the high CrCP values (the pressure-intercept) and changes with tasks. It is interesting to see that, although the PI is often used as a measure of cerebrovascular resistance, ${\mathrm{CVR}}_{i}$ and PI do not always match during task conditions, likely because the PI is also affected by the compliance of the cerebral arterial bed, ABP, and HR,^57^ which differentially vary during the selected tasks.

As shown in Fig. 2, this method allows for the separation of inflow and outflow contributions to the pulsatile flow. The separation of the individual contributions may be useful when trying to estimate intracranial pressure using pulsatile features because increases in ICP would likely affect the lower-pressure venous outflow more than, and in a different way than, the higher-pressure arterial inflow.

With respect to the slow hemodynamic signal’s changes measured during the various tasks, our findings are in agreement with what was expected based on literature reports. Hemoglobin concentration changes during HV^58^^,^^59^ and BH^60^ are consistent with previous NIRS findings. The ${\mathrm{CBF}}_{i}$ response during HV and BH is consistent with blood flow results previously reported by our group.^17^ The decreases in ${\mathrm{CVR}}_{i}$ and PI during BH are consistent with the findings of TCD ultrasound studies.^61^^–^^63^ During the cold pressor tasks, the measured increases in CBF, hemoglobin oxygenation, and total hemoglobin concentration are due to systemic blood pressure increases above cerebral autoregulation thresholds and, in the frontal area, are also due to functional activity caused by pain stimulation.^64^ CBF increases during CPT were previously observed with TCD,^65^ and cerebral oxygenation increase aligns with prior NIRS studies.^66^^–^^68^ Similarly, during cold pressor tasks, decreases in PI have been observed with TCD,^69^^,^^70^ and the increases that we observed in ${\mathrm{CVR}}_{i}$ are consistent with TCD-derived resistance area product (RAP) increases reported by Panerai et al.^71^

This study has several limitations. Because of optical crosstalk, we could not measure NIRS and DCS in the same location, but instead measured them contralaterally across the forehead. This may account for the small differences that we observed between the ${\mathrm{pCBF}}_{i}$ and ${\mathrm{pCBF}}_{i\text{-}\text{fit}}$ waveforms. This problem can be resolved in the future by unifying the NIRS and DCS systems using a single probe and temporal multiplexing by pulsing the DCS laser source.

We assumed a homogenous medium. To further separate the scalp and brain signals, the addition of multi-layer models for pulsatile NIRS and DCS is required.

The NIRS-PPG fitting procedure is affected by having several local minima, especially for the NIRS-PPG contribution term $C_{2}$. This is because the NIRS-PPG waveform has fewer distinct features and contributes less than the first derivative. We overcame this problem by grid-searching for the initial guess.

To demonstrate the method, we used a high-end DCS device with a more than 16 times higher SNR than conventional DCS. When using conventional DCS at traditional NIRS wavelengths, the recovered ${\mathrm{pCBF}}_{i}$ will be much noisier. With current systems, we can use larger pulse averaging and shorter source-detector separations to reduce the errors in determining the $C_{1}$ and $C_{2}$ fitting parameters. Novel DCS devices are emerging with improved SNR, comparable to the one of the SNSPD-DCS system used here, for which shorter averaging should be sufficient.

Cardiac-gated averaging introduces error, especially when the HR varies. Using the combined NIRS-DCS approach, we were able to substantially reduce the number of cardiac-gated averaging needed to obtain clean pulsatile blood flow signals.

In conclusion, the use of NIRS-PPG and d(NIRS-PPG)/dt allows for achieving pulsatile blood flow with high temporal resolution and minimal cardiac-gated averaging. Combined with DCS, to obtain calibration, it allows us to derive CrCP and ${\mathrm{CVR}}_{i}$ at a rate of 4 s. The combination of FlexNIRS and SNSPD-DCS at 1064 nm allows for the comprehensive probing of hemodynamic response during breathing and other tasks.

## Code, Data, and Materials

De-identified data will be provided upon request to the authors following MGB regulations. FlexNIRS is an open-source NIRS cerebral oximeter that can be used for research purposes. Information can be found on our website.

## References

[1] Wray S. et al., “Characterization of the near infrared absorption spectra of cytochrome aa3 and haemoglobin for the non-invasive monitoring of cerebral oxygenation,” Biochim. Biophys. Acta Bioenerg. 933(1), 184–192 (1988). 10.1016/0005-2728(88)90069-2 2831976

[2] Jöbsis F. F., “Noninvasive, infrared monitoring of cerebral and myocardial oxygen sufficiency and circulatory parameters,” Science 198(4323), 1264–1267 (1977). SCIEAS 0036-8075 10.1126/science.929199 929199

[3] Boas D. A. Yodh A. G., “Spatially varying dynamical properties of turbid media probed with diffusing temporal light correlation,” JOSA A 14(1), 192–215 (1997). 10.1364/JOSAA.14.000192

[r4] Buckley E. M. et al., “Diffuse correlation spectroscopy for measurement of cerebral blood flow: future prospects,” Neurophotonics 1(1), 011009 (2014). 10.1117/1.NPh.1.1.011009 25593978 PMC4292799

[5] Mesquita R. C. et al., “Direct measurement of tissue blood flow and metabolism with diffuse optics,” Philos. Trans. R. Soc. A 369(1955), 4390–4406 (2011). PTRMAD 1364-503X 10.1098/rsta.2011.0232 PMC3263785 22006897

[6] Boas D. A. Franceschini M. A., “Haemoglobin oxygen saturation as a biomarker: the problem and a solution,” Philos. Trans. R. Soc. A Math. Phys. Eng. Sci. 369(1955), 4407–4424 (2011). 10.1098/rsta.2011.0250 PMC3263786 22006898

[7] Durduran T. et al., “Diffuse optical measurement of blood flow, blood oxygenation, and metabolism in a human brain during sensorimotor cortex activation,” Opt. Lett. 29(15), 1766–1768 (2004). OPLEDP 0146-9592 10.1364/OL.29.001766 15352363

[8] Roche-Labarbe N. et al., “Somatosensory evoked changes in cerebral oxygen consumption measured non-invasively in premature neonates,” Neuroimage 85, 279–286 (2014). NEIMEF 1053-8119 10.1016/j.neuroimage.2013.01.035 23370052 PMC3686986

[9] Dehaes M. et al., “Cerebral oxygen metabolism in neonatal hypoxic ischemic encephalopathy during and after therapeutic hypothermia,” J. Cereb. Blood Flow Metab. 34(1), 87–94 (2014). 10.1038/jcbfm.2013.165 24064492 PMC3887346

[10] Zavriyev A. I. et al., “The role of diffuse correlation spectroscopy and frequency-domain near-infrared spectroscopy in monitoring cerebral hemodynamics during hypothermic circulatory arrests,” JTCVS Tech. 7, 161–177 (2021). 10.1016/j.xjtc.2021.01.023 34318236 PMC8311503

[11] Wu K.-C. et al., “Validation of diffuse correlation spectroscopy measures of critical closing pressure against transcranial Doppler ultrasound in stroke patients,” J. Biomed. Opt. 26(3), 036008 (2021). JBOPFO 1083-3668 10.1117/1.JBO.26.3.036008 33774980 PMC7998065

[r12] Baker W. B. et al., “Noninvasive optical monitoring of critical closing pressure and arteriole compliance in human subjects,” J. Cereb. Blood Flow Metab. 37(8), 2691–2705 (2017). 10.1177/0271678X17709166 28541158 PMC5536813

[r13] Ruesch A. et al., “Estimating intracranial pressure using pulsatile cerebral blood flow measured with diffuse correlation spectroscopy,” Biomed. Opt. Express 11(3), 1462–1476 (2020). BOEICL 2156-7085 10.1364/BOE.386612 32206422 PMC7075623

[r14] Relander F. A. J. et al., “Using near-infrared spectroscopy and a random forest regressor to estimate intracranial pressure,” Neurophotonics 9(4), 045001 (2022). 10.1117/1.NPh.9.4.045001 36247716 PMC9552940

[r15] Fischer J. B. et al., “Non-invasive estimation of intracranial pressure by diffuse optics: a proof-of-concept study,” J. Neurotrauma 37(23), 2569–2579. JNEUE4 0897-7151 10.1089/neu.2019.6965 32460617

[16] Lafontant A. et al., “Comparison of optical measurements of critical closing pressure acquired before and during induced ventricular arrhythmia in adults,” Neurophotonics 9(3), 035004 (2022). 10.1117/1.NPh.9.3.035004 36039170 PMC9407009

[17] Ozana N. et al., “Superconducting nanowire single-photon sensing of cerebral blood flow,” Neurophotonics 8(3), 035006 (2021). 10.1117/1.NPh.8.3.035006 34423069 PMC8373637

[18] Early C. B. et al., “Dynamic pressure-flow relationships of brain blood flow in the monkey,” J. Neurosurg. 41(5), 590–596 (1974). JONSAC 0022-3085 10.3171/jns.1974.41.5.0590 4422124

[19] Selb J. et al., “Sensitivity of near-infrared spectroscopy and diffuse correlation spectroscopy to brain hemodynamics: simulations and experimental findings during hypercapnia,” Neurophotonics 1(1), 015005 (2014). 10.1117/1.NPh.1.1.015005 25453036 PMC4247161

[20] Zhou C. et al., “Diffuse optical correlation tomography of cerebral blood flow during cortical spreading depression in rat brain,” Opt. Express 14(3), 1125 (2006). OPEXFF 1094-4087 10.1364/OE.14.001125 19503435

[21] Strangman G. E. et al., “Increased cerebral blood volume pulsatility during head-down tilt with elevated carbon dioxide: the SPACECOT study,” J. Appl. Physiol. 123(1), 62–70 (2017). 10.1152/japplphysiol.00947.2016 28360122

[22] Wu K.-C. et al., “Open-source FlexNIRS: a low-cost, wireless and wearable cerebral health tracker,” Neuroimage 256, 119216 (2022). NEIMEF 1053-8119 10.1016/j.neuroimage.2022.119216 35452803 PMC11262416

[23] Kyriacou P. A. Allen J., Photoplethysmography: Technology, Signal Analysis and Applications, Academic Press (2021).

[24] Millasseau S. C. et al., “Determination of age-related increases in large artery stiffness by digital pulse contour analysis,” Clin. Sci. 103(4), 371–377 (2002). 10.1042/cs1030371 12241535

[r25] Allen J. Murray A., “Age-related changes in the characteristics of the photoplethysmographic pulse shape at various body sites,” Physiol. Meas. 24(2), 297–307 (2003). PMEAE3 0967-3334 10.1088/0967-3334/24/2/306 12812416

[r26] Yousef Q. Reaz M. B. I. Ali M. A. M., “The analysis of PPG morphology: investigating the effects of aging on arterial compliance,” Meas. Sci. Rev. 12(6), 266–271 (2012). 10.2478/v10048-012-0036-3

[27] Park J. et al., “Photoplethysmogram analysis and applications: an integrative review,” Front. Physiol. 12, 808451 (2022). FROPBK 0301-536X 10.3389/fphys.2021.808451 35300400 PMC8920970

[28] Fabiani M. et al., “Taking the pulse of aging: mapping pulse pressure and elasticity in cerebral arteries with optical methods,” Psychophysiology 51(11), 1072–1088 (2014). PSPHAF 0048-5772 10.1111/psyp.12288 25100639 PMC9906973

[r29] Chiarelli A. M. et al., “Individual differences in regional cortical volumes across the life span are associated with regional optical measures of arterial elasticity,” Neuroimage 162, 199–213 (2017). NEIMEF 1053-8119 10.1016/j.neuroimage.2017.08.064 28866349 PMC5705573

[30] Mohammadi H. et al., “Longitudinal impact of physical activity on brain pulsatility index and cognition in older adults with cardiovascular risk factors: a NIRS study,” Brain Sci. 11(6), 730 (2021). 10.3390/brainsci11060730 34072651 PMC8230110

[31] Cook L. B., “Extracting arterial flow waveforms from pulse oximeter waveforms: apparatus,” Anaesthesia 56(6), 551–555 (2001). 10.1046/j.1365-2044.2001.01986.x 11412161

[r32] Wisely N. A. Cook L. B., “Arterial flow waveforms from pulse oximetry compared with measured Doppler flow waveforms: apparatus,” Anaesthesia 56(6), 556–561 (2001). 10.1046/j.1365-2044.2001.01987.x 11412162

[33] Themelis G. et al., “Near-infrared spectroscopy measurement of the pulsatile component of cerebral blood flow and volume from arterial oscillations,” J. Biomed. Opt. 12(1), 014033 (2007). JBOPFO 1083-3668 10.1117/1.2710250 17343508 PMC2637815

[34] Siegert A. J. F., On the Fluctuations in Signals Returned by Many Independently Moving Scatterers, Radiation Laboratory, Massachusetts Institute of Technology (1943).

[35] Hines E. A., “A clinical test of vasomotor irritablilty: blood pressure response to cold,” (1933).

[36] Hueber D. M. et al., “New optical probe designs for absolute (self-calibrating) NIR tissue hemoglobin measurements,” Proc. SPIE 3597, 618–631 (1999). PSISDG 0277-786X 10.1117/12.356784

[37] Blaney G. et al., “Broadband absorption spectroscopy in tissue: combining dual-slope continuous-wave and self-calibrating frequency-domain measurements,” Proc. SPIE 11639, 68–74 (2021). PSISDG 0277-786X 10.1117/12.2582792

[38] Hallacoglu B. et al., “Absolute measurement of cerebral optical coefficients, hemoglobin concentration and oxygen saturation in old and young adults with near-infrared spectroscopy,” J. Biomed. Opt. 17(8), 081406 (2012). JBOPFO 1083-3668 10.1117/1.JBO.17.8.081406 23224167 PMC3412596

[39] Fatouros P. P. Marmarou A., “Use of magnetic resonance imaging for in vivo measurements of water content in human brain: method and normal values,” J. Neurosurg. 90(1), 109–115 (1999). JONSAC 0022-3085 10.3171/jns.1999.90.1.0109 10413163

[40] Boas D. A. Campbell L. E. Yodh A. G., “Scattering and imaging with diffusing temporal field correlations,” Phys. Rev. Lett. 75(9), 1855 (1995). PRLTAO 0031-9007 10.1103/PhysRevLett.75.1855 10060408

[41] Carp S. A. et al., “Diffuse correlation spectroscopy measurements of blood flow using 1064 nm light,” J. Biomed. Opt. 25(9), 097003 (2020). JBOPFO 1083-3668 10.1117/1.JBO.25.9.097003 32996299 PMC7522668

[42] Delpy D. T. et al., “Estimation of optical pathlength through tissue from direct time of flight measurement,” Phys. Med. Biol. 33(12), 1433–1442 (1988). PHMBA7 0031-9155 10.1088/0031-9155/33/12/008 3237772

[43] Durduran T. Yodh A. G., “Diffuse correlation spectroscopy for non-invasive, micro-vascular cerebral blood flow measurement,” Neuroimage 85, 51–63 (2014). NEIMEF 1053-8119 10.1016/j.neuroimage.2013.06.017 23770408 PMC3991554

[44] Du Le V. N. Srinivasan V. J., “Beyond diffuse correlations: deciphering random flow in time-of-flight resolved light dynamics,” Opt. Express 28(8), 11191–11214 (2020). OPEXFF 1094-4087 10.1364/OE.385202 32403635 PMC7340374

[45] Boas D. A. et al., “Establishing the diffuse correlation spectroscopy signal relationship with blood flow,” Neurophotonics 3(3), 031412 (2016). 10.1117/1.NPh.3.3.031412 27335889 PMC4904065

[46] Gavin H. P., The Levenberg-Marquardt Algorithm for Nonlinear Least Squares Curve-Fitting Problems, p. 19, Department of Civil and Environmental Engineering, Duke University (2019).

[47] De Riva N. et al., “Transcranial Doppler pulsatility index: what it is and what it isn’t,” Neurocrit. Care 17(1), 58–66 (2012). 10.1007/s12028-012-9672-6 22311229

[48] Bellner J. et al., “Transcranial Doppler sonography pulsatility index (PI) reflects intracranial pressure (ICP),” Surg. Neurol. 62(1), 45–51 (2004). SGNRAI 0090-3019 10.1016/j.surneu.2003.12.007 15226070

[49] Raichle M. E. Plum F., “Hyperventilation and cerebral blood flow,” Stroke 3(5), 566–575 (1972). SJCCA7 0039-2499 10.1161/01.STR.3.5.566 4569138

[50] Gouvea Bogossian E. et al., “Hyperventilation in adult TBI patients: how to approach it?” Front. Neurol. 11, 580859 (2021). 10.3389/fneur.2020.580859 33584492 PMC7875871

[51] Wilson D. F. et al., “Effect of hyperventilation on oxygenation of the brain cortex of newborn piglets,” J. Appl. Physiol. 70(6), 2691–2696 (1991). 10.1152/jappl.1991.70.6.2691 1909316

[52] Parkes M. J. et al., “Assessing and ensuring patient safety during breath-holding for radiotherapy,” Br. J. Radiol. 87(1043), 20140454 (2014). BJRAAP 0007-1285 10.1259/bjr.20140454 25189121 PMC4207152

[53] Perini R. et al., “Heart rate and blood pressure time courses during prolonged dry apnoea in breath-hold divers,” Eur. J. Appl. Physiol. 104, 1–7 (2008). EJAPFN 1439-6319 10.1007/s00421-008-0771-1 18496707

[54] Zerweck L. et al., “Investigation of the BOLD-based MRI signal time course during short breath-hold periods for estimation of the cerebrovascular reactivity,” SN Compr. Clin. Med. 2(9), 1551–1562 (2020). 10.1007/s42399-020-00442-6

[55] Larsen J. et al., “Breath holding for 20 s following extended expiration is a practical, effective and robust standard when measuring cerebrovascular reactivity in healthy adults using BOLD fMRI at 3 T,” Neuroimage Rep. 1(2), 100021 (2021). 10.1016/j.ynirp.2021.100021 PMC12172711 40567865

[56] Davies H. J. et al., “In-ear spo2: a tool for wearable, unobtrusive monitoring of core blood oxygen saturation,” Sensors 20(17), 4879 (2020). SNSRES 0746-9462 10.3390/s20174879 32872310 PMC7506719

[57] Varsos G. V. et al., “A noninvasive estimation of cerebral perfusion pressure using critical closing pressure,” J. Neurosurg. 123, 638–648 (2015). JONSAC 0022-3085 10.3171/2014.10.JNS14613 25574566

[58] Sandru S. et al., “Near-infrared spectroscopy usefulness in validation of hyperventilation test,” Medicina 58(10), 1396 (2022). 10.3390/medicina58101396 36295560 PMC9607377

[59] Matsuo K. et al., “Prefrontal hemodynamic response to verbal-fluency task and hyperventilation in bipolar disorder measured by multi-channel near-infrared spectroscopy,” J. Affect. Disord. 82(1), 85–92 (2022). JADID7 10.1016/j.jad.2003.10.004 15465580

[60] Raitamaa L. et al., “Breath hold effect on cardiovascular brain pulsations–a multimodal magnetic resonance encephalography study,” J. Cereb. Blood Flow Metab. 39(12), 2471–2485 (2019). 10.1177/0271678X18798441 30204040 PMC6893986

[61] van Veen T. R. et al., “Effect of breath holding on cerebrovascular hemodynamics in normal pregnancy and preeclampsia,” J. Appl. Physiol. 118(7), 858–862 (2015). 10.1152/japplphysiol.00562.2014 25614597

[r62] Settakis G. et al., “Transcranial Doppler study of the cerebral hemodynamic changes during breath-holding and hyperventilation tests,” J. Neuroimaging 12(3), 252–258 (2002). JNERET 1051-2284 10.1111/j.1552-6569.2002.tb00129.x 12116744

[63] Hsu H.-Y. et al., “Correlations among critical closing pressure, pulsatility index and cerebrovascular resistance,” Ultrasound Med. Biol. 30(10), 1329–1335 (2004). USMBA3 0301-5629 10.1016/j.ultrasmedbio.2004.08.006 15582232

[64] La Cesa S. et al., “fMRI pain activation in the periaqueductal gray in healthy volunteers during the cold pressor test,” Magn. Reson. Imaging 32(3), 236–240 (2014). MRIMDQ 0730-725X 10.1016/j.mri.2013.12.003 24468081

[65] Flück D. et al., “Extra-and intracranial blood flow regulation during the cold pressor test: influence of age,” J. Appl. Physiol. 123(5), 1071–1080 (2017). 10.1152/japplphysiol.00224.2017 28663374 PMC5792099

[66] Barati Z. et al., “Hemodynamic response to repeated noxious cold pressor tests measured by functional near infrared spectroscopy on forehead,” Ann. Biomed. Eng. 41(2), 223–237 (2013). ABMECF 0090-6964 10.1007/s10439-012-0642-0 22956158

[r67] Høiseth L. Ø. et al., “Tissue oxygen saturation and finger perfusion index in central hypovolemia: influence of pain,” Crit. Care Med. 43(4), 747–756 (2015). CCMDC7 0090-3493 10.1097/CCM.0000000000000766 25513787

[68] Barati Z. Zakeri I. Pourrezaei K., “Functional near-infrared spectroscopy study on tonic pain activation by cold pressor test,” Neurophotonics 4(1), 015004 (2017). 10.1117/1.NPh.4.1.015004 28386576 PMC5358549

[69] Tsai S.-J. et al., “Impairment of cerebral hemodynamic response to the cold pressor test in patients with Parkinson’s disease,” Parkinsonism Relat. Disord. 15(2), 94–100 (2009). 10.1016/j.parkreldis.2008.03.007 18440850

[70] Micieli G. et al., “Increased cerebral blood flow velocity induced by cold pressor test in migraine: a possible basis for pathogenesis?” Cephalalgia 15(6), 494–498 (1995). CEPHDF 0333-1024 10.1046/j.1468-2982.1995.1506494.x 8706113

[71] Panerai R. B. et al., “Cerebral blood flow velocity response to induced and spontaneous sudden changes in arterial blood pressure,” Am. J. Physiol. Circ. Physiol. 280(5), H2162–H2174 (2001). 10.1152/ajpheart.2001.280.5.H2162 11299218

