# Supplementary material for: Enhancing diffuse correlation spectroscopy pulsatile cerebral blood flow signal with near-infrared spectroscopy photoplethysmography
Source: Neurophotonics. 2023 Sep 6;10(3):035008. doi: 10.1117/1.NPh.10.3.035008 (PMC10482352; doi:10.1117/1.NPh.10.3.035008)
Supplement: Supplementary file 1 [file NPh_010_035008_SD001.pdf]

Supplementary

Table S1. Participant demographic information.

New	Age	Gender	BMI	Height cm	Weight kg	Skin tone
**sub01**	23	Male	20.0	168	56	Light
**sub02**	24	Male	28.2	170	82	Light
**sub03**	63	Female	25.1	173	75	Light
**sub04**	68	Male	29.6	170	86	Light
**sub05**	40	Male	19.5	180	63	Light
**sub06**	23	Female	22.9	157	57	Dark
**sub07**	36	Male	24.3	165	66	Medium
**sub08**	55	Female	28.2	170	82	Dark
**sub09**	26	Female	25.4	158	64	Light
**sub10**	21	Male	25.8	168	73	Light
**Avg ± SD**	38±17	4F, 6M	24.9±3.2	167.9±6.4	70.4±10.2	7 L, 1 M, 2 D

For the skin tone classification, we used the Fitzpatrick classification of skin types regrouped as: Light: pale white skin, white fair skin (types 1 and 2); Medium: medium white skin, olive, light brown skin (types 3 and 4); Dark: brown skin, dark brown, or black skin (types 5 and 6).

(L: light, M: medium, D: dark)

Abbreviations

ABP arterial blood pressure
BF_i blood flow index
BF_{in} pulsatile blood inflow
BF_{out} pulsatile blood outflow
BH breath-holding
CBF cerebral blood flow
CBF_i cerebral blood flow index
CPT cold pressor test
CrCP critical closing pressure
CV coefficient of variation
CVR_i cerebrovascular resistance index
d(NIRS-PPG)/dt first time derivative of NIRS-PPG
DCS diffuse correlation spectroscopy
DN dicrotic notch
DP diastolic peak
ECG electrocardiogram
FPGA field-programmable gate array
g_2 intensity temporal autocorrelation function
Hb hemoglobin concentration
HbO oxyhemoglobin concentration
HbR deoxyhemoglobin concentration
HbT total hemoglobin concentration
HR heart rate
HV hyperventilation
IQR interquartile range
MAP mean arterial blood pressure
μ_a absorption coefficient
μ_s' reduced scattering coefficient
NEP noise equivalent power
NIRS near-infrared spectroscopy
NIRS-PPG photoplethysmography at large source-detector separations
P1 systolic peak
P2 secondary systolic peak
pABP pulsatile arterial blood pressure
$pCBF_i$ pulsatile cerebral blood flow index
$pCBF_{i\text{-}fit}$ pulsatile cerebral blood flow index obtained through fitting NIRS-PPG
PI pulsatility index
PPG photoplethysmography
PWA pulse waveform analysis
SDsep source-detector separation
SNR signal-to-noise ratio
SNSPD superconducting nanowire single-photon detector
SO_2 hemoglobin oxygen saturation
SpO_2 peripheral oxygen saturation
TCD transcranial Doppler ultrasound

Multi-distance method issues in the presence of inhomogeneities

FlexNIRS uses a self-calibrating multi-distance method to quantify hemoglobin concentration and oxygenation. The data acquisition software has built-in criteria that assess in real-time the quality of the probe positioning and alerts the users for asymmetries across the two sets of separations if differences are above the preset thresholds. Because these experiments required bilateral symmetric positioning of FlexNIRS and DCS probes, in 2 subjects, #4 and #5, we could not find a position with acceptable FlexNIRS symmetry. Hence for these two subjects, we used the average hemoglobin concentration measured across the other 8 subjects (HbO of 51.5 μM, HbR of 29.8 μM, and SO_2 of 63%). We show here how the effect of asymmetry caused by tissue inhomogeneities can result in highly erroneous multi-distance SO_2 and Hb estimates.

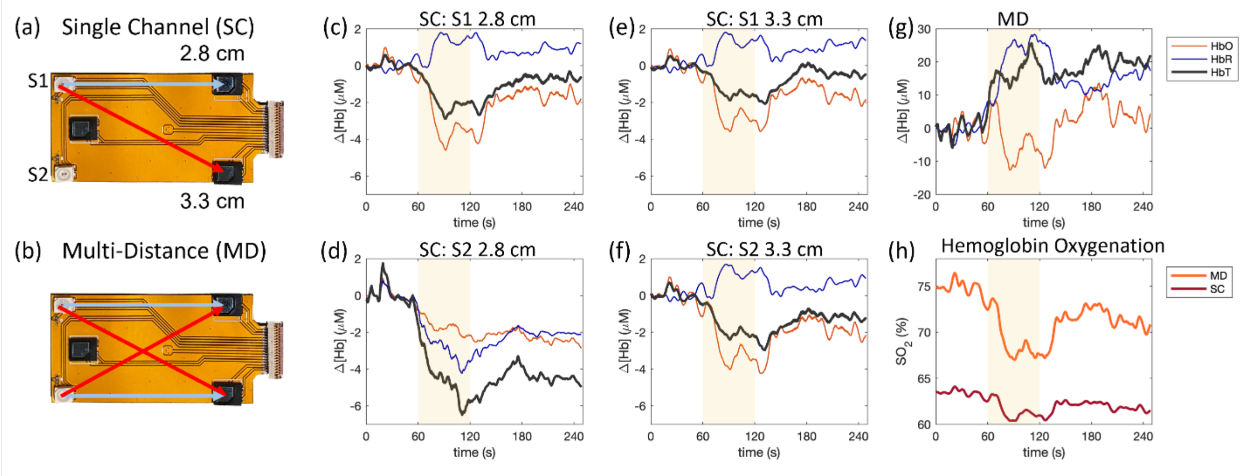

Fig. S1. Single channel (SC) and multi-distance (MD) changes in hemoglobin concentration and oxygenation measured in subject #5 during hyperventilation (yellow area). Panels (a) and (b) show the probe geometry. For the single channel calculation of changes in hemoglobin concentrations, the four source-detector pairs are used independently (a); for the multi-distance method, the four source-detector pairs are combined (b) and the MD reflectance slopes (slope of $\ln(\rho^2 R)$ vs. ρ; ρ: SDsep, R: reflectance) between the two distances (self-calibrating geometry) are used to estimate absorption coefficients and to derive hemoglobin concentrations. Panels (c) to (f): Hemoglobin concentration changes calculated from the four individual LED-photodetector pairs, single channel method. In panel (d), hemoglobin concentration changes differ substantially from the changes in the other three positions (panels (c), (e), and (f)), probably due to a large superficial blood vessel between S2 and the closer detector (2.8 cm SDsep). This affects the MD reflectance slope calculations in the multi-distance method, leading to an overestimation of absorption coefficients, with μ_a 0.5-0.8 cm^{-1} vs. the typical 0.1-0.2 cm^{-1}, an overestimation of hemoglobin concentration both absolute (HbT: 270 mM vs. the other 8 subjects 81 mM) and changes (ΔHbR: 30 mM vs. SC 2 mM) (panel (g)). It also affects the direction of the changes of total hemoglobin concentration with hyperventilation, showing an apparent vasodilation instead of the expected vasoconstriction. Consequently, the derived hemoglobin oxygen saturation (panel (h), orange) is higher and with larger changes than expected. In panel (h), we also report the recovered SO_2 assuming initial hemoglobin concentration from the average baseline of the other 8 subjects and calculated using a single channel, in this case SC S1 at 3.3 cm (dark red).

(SC: single channel, MD: multi-distance, S1 and S2: source 1 and source 2, HbR: deoxyhemoglobin, HbO: oxyhemoglobin, HbT: total hemoglobin, LED: light-emitting diode, SDsep: source-detector separation, μ_{a}: absorption coefficient, SO_2: hemoglobin oxygen saturation)

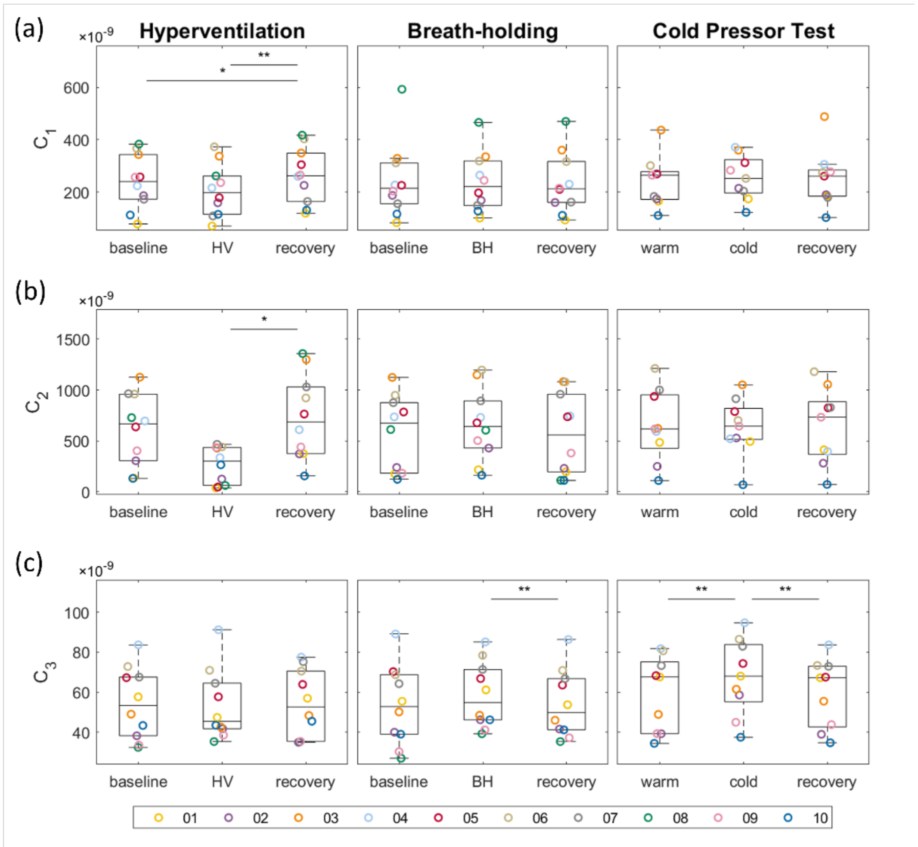

Fig. S2. (a) to (c) Fitting parameters C_1, C_2 and C_3 across subjects and tasks. The asterisks indicate paired-sample sign test significant difference levels. (*: $p < 0.05$, **: $p < 0.01$, paired-sample sign test)

(C_1, C_2 and C_3 are the fitting parameters in equation (4))

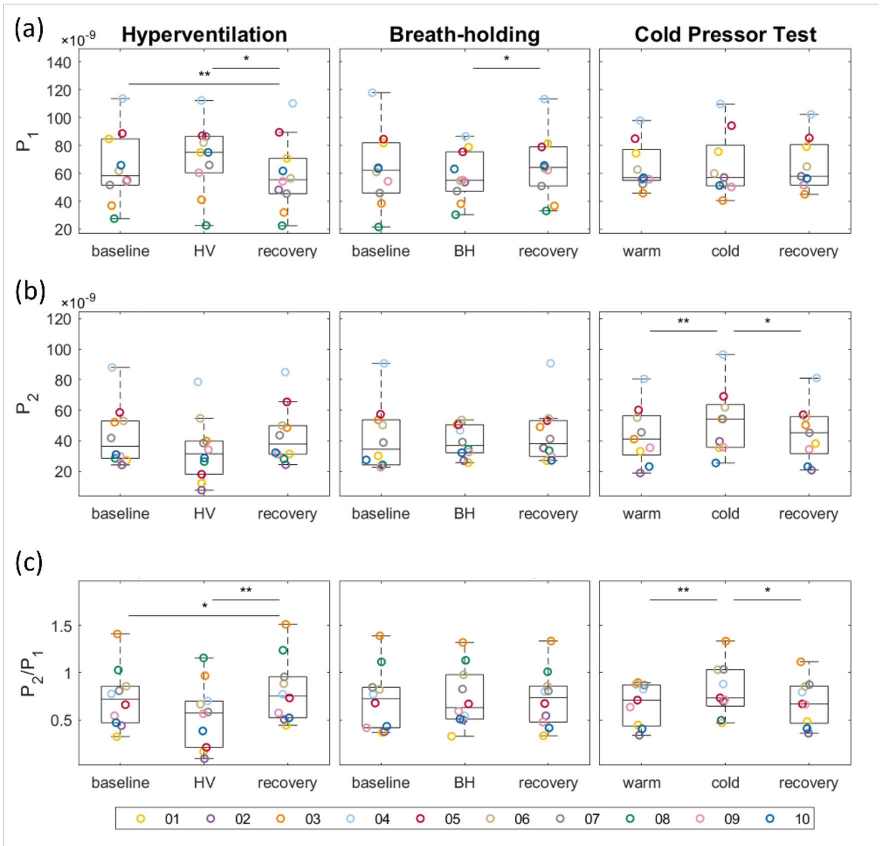

Fig. S3. PWA peaks extracted from pCBF$_{i\text{-}fit}$. The amplitude of the peaks was calculated relative to the diastolic blood flow. Each color represents a subject as indicated in the legend. The asterisks indicate paired-sample sign test significant difference levels. (a) Systolic peak P_1 and (b) the secondary systolic peak P_2, and (c) the ratios between the two peaks across subjects and tasks. (*: $p < 0.05$, **: $p < 0.01$, paired-sample sign test)

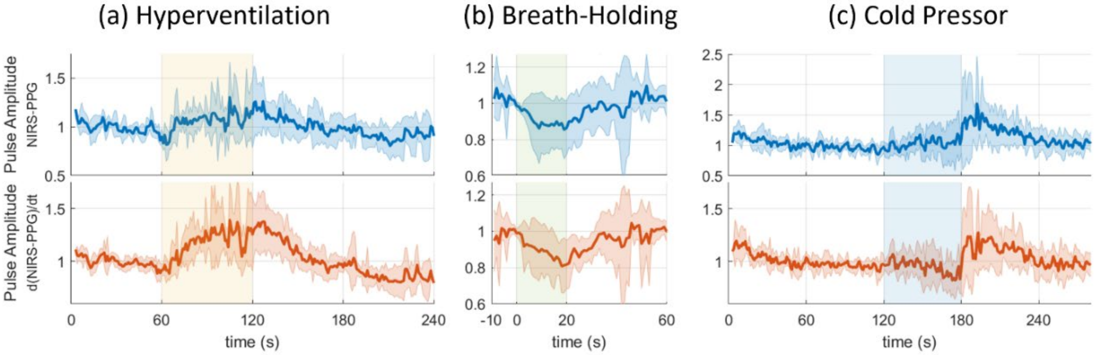

Fig. S4. Group average of pulse amplitude of NIRS-PPG (top panels) and d(NIRS-PPG) /dt (bottom panels) during (a) hyperventilation task (n=8, HV period in yellow), (b) breath-holding task (n=8, block average 4 repetitions, BH period in green) and (c) cold pressor test (n=7, CPT period in cyan). Standard deviation across subjects shown in lighter color.

(NIRS-PPG: near-infrared spectroscopy photoplethysmography, HV: hyperventilation, BH: breath-holding, CPT: cold pressor test,)
